# The role of altered protein acetylation in neurodegenerative disease

**DOI:** 10.3389/fnagi.2022.1025473

**Published:** 2023-01-04

**Authors:** Fariha Kabir, Rachel Atkinson, Anthony L. Cook, Andrew James Phipps, Anna Elizabeth King

**Affiliations:** Wicking Dementia Research and Education Center, College of Health and Medicine, University of Tasmania, Hobart, TAS, Australia

**Keywords:** PTMs, acetylation, HDACs, HATs, proteostasis, cytoskeleton, neurodegenerative disease

## Abstract

Acetylation is a key post-translational modification (PTM) involved in the regulation of both histone and non-histone proteins. It controls cellular processes such as DNA transcription, RNA modifications, proteostasis, aging, autophagy, regulation of cytoskeletal structures, and metabolism. Acetylation is essential to maintain neuronal plasticity and therefore essential for memory and learning. Homeostasis of acetylation is maintained through the activities of histone acetyltransferases (HAT) and histone deacetylase (HDAC) enzymes, with alterations to these tightly regulated processes reported in several neurodegenerative diseases including Alzheimer’s disease (AD), Parkinson’s disease (PD), Huntington’s disease (HD), and amyotrophic lateral sclerosis (ALS). Both hyperacetylation and hypoacetylation can impair neuronal physiological homeostasis and increase the accumulation of pathophysiological proteins such as tau, α-synuclein, and Huntingtin protein implicated in AD, PD, and HD, respectively. Additionally, dysregulation of acetylation is linked to impaired axonal transport, a key pathological mechanism in ALS. This review article will discuss the physiological roles of protein acetylation and examine the current literature that describes altered protein acetylation in neurodegenerative disorders.

## Introduction

Post-translational modifications (PTMs) of proteins define the molecular complexity of our cells. Through mechanisms such as covalent modifications of proteins, PTMs change the properties of a protein to determine its activity, localization, or interaction with other proteins, cells, or systems (Mann and Jensen, 2003). Over 400 PTMs have been reported in the literature, including methylation, acetylation, ubiquitination, phosphorylation, and glycosylation (Duan and Walther, 2015), which change the function of the proteins, leading to altered gene expression, cellular signaling, protein trafficking, and cellular structure (Khoury et al., 2011; Duan and Walther, 2015). Given the importance of PTMs, our understanding of the role of these modifications in complex neurodegenerative diseases is critical to understanding the causes and finding treatments or cures.

Age-related neurodegenerative diseases, including Alzheimer’s disease (AD), Huntington’s disease (HD), prion diseases, Parkinson’s disease (PD), frontotemporal lobar degeneration (FTLD), and amyotrophic lateral sclerosis (ALS) are some of the leading causes of mortality and morbidity worldwide (Taylor et al., 2002; Erkkinen et al., 2018; Ashby, 2019). These diseases are progressive and heterogeneous in nature and involve adverse changes to the central and/or peripheral nervous system, including the degeneration of neurons resulting in the loss of cognitive and/or motor functions (Lin and Beal, 2006; Hrelia et al., 2020). Due to the impact of neurodegenerative disease, developing targeted treatments based on pathological mechanisms has become a major focus of research. Aggregation of misprocessed proteins is a common feature of the major types of neurodegenerative disorders (Merlini et al., 2001; Tutar et al., 2013; Hrelia et al., 2020), which can be predisposed by genetic mutations, but the majority of cases are of unknown etiology, or sporadic in nature (Tutar et al., 2013). We may be able to discern the driving factors behind neurodegenerative disease by understanding how PTMs of disease-associated proteins alter underlying pathological processes, through mechanisms such as impaired gene regulation, proteostasis, or alterations to the cytoskeleton.

The role of altered PTMs in neurodegenerative disease is highlighted by findings that many disease-associated aggregated proteins have abnormal PTMs, which can result in protein aggregation, mislocalization, and/or misprocessing (Schaffert and Carter, 2020). Such dysregulation may ultimately be linked to downstream pathogenesis such as glutamate excitotoxicity, mitochondrial dysfunction, and activation of caspases (Hall, 2011), suggesting that altered PTMs may be drivers of neurodegeneration, and potential targets for novel therapeutic strategies. Studies to date have predominantly focused on the role of altered phosphorylation in neurodegenerative diseases (Hanger et al., 2009; Rudrabhatla, 2014), however, alterations in acetylation, particularly of cytoskeletal proteins, may also play a role in driving neurodegeneration (Sternberger et al., 1985; Cavallarin et al., 2010; Noble et al., 2013; Rudrabhatla, 2014; Xu et al., 2015; Schaffert and Carter, 2020). This review will provide a general overview of the role of protein acetylation and how it can be dysregulated in neurodegenerative disease. As this is a vast topic, we will focus on the disease-associated proteins and the cytoskeleton.

## Acetylation

Acetylation is a key biological process for regulating the function and viability of all mammalian cells, by adding acetyl groups to the structure of proteins. Acetylation can occur on different chemical groups including hydroxyl, thiol, or amino groups (Portaleone, 2004). Although acetylation was first identified as a modification of histone proteins within the nucleus, it also occurs on several non-histone proteins, where it plays an essential role in regulating cellular responses and signaling in response to different types of stressors, both internal and external (Drazic et al., 2016).

**Figure 1:**
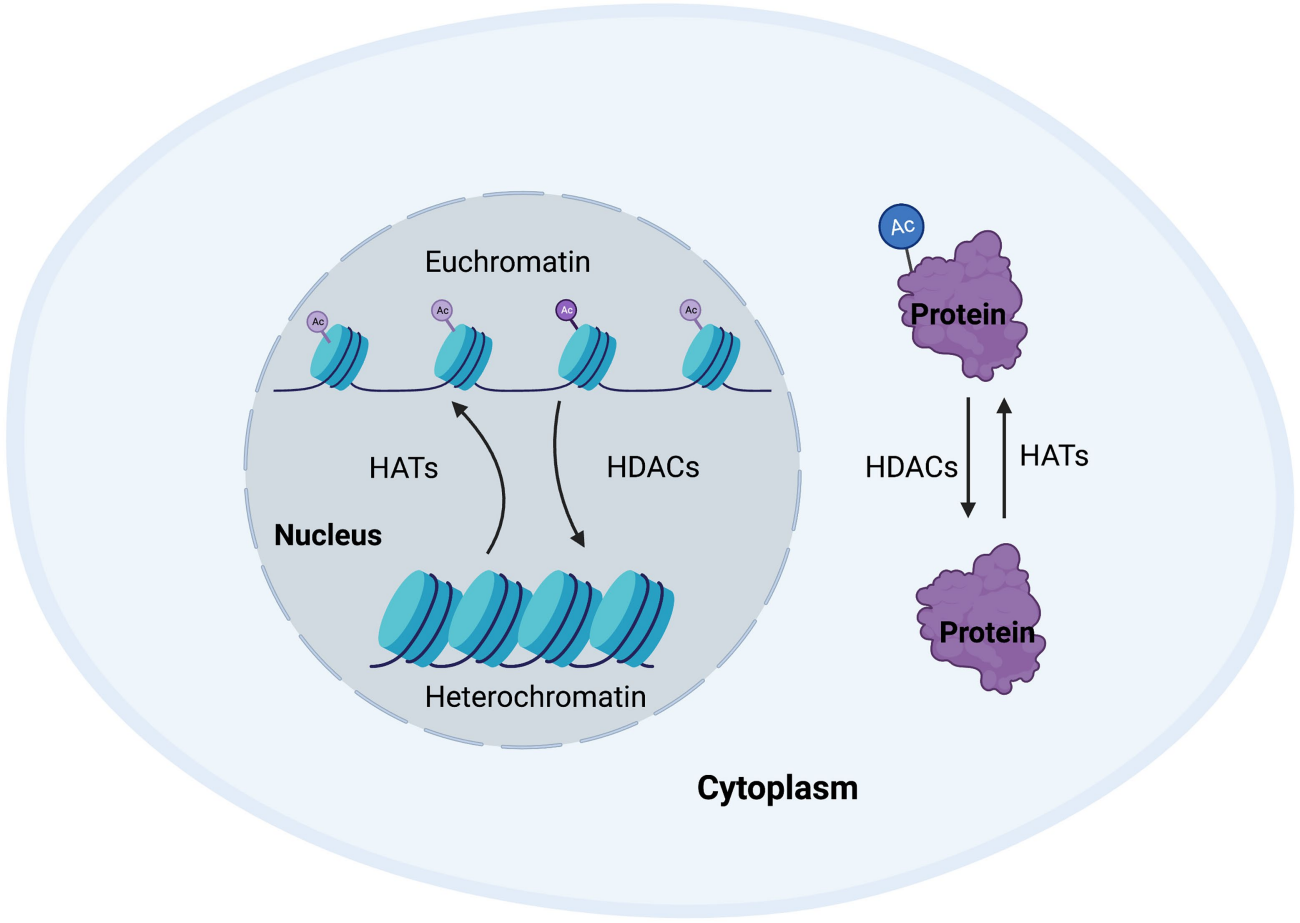
Cellular homeostasis regulated by histone acetyltransferases (HATs) and histone deacetylases (HDACs). Post-translational modifications regulate a wide range of proteins to enable appropriate transcription and maintain cellular function. HATs and HDACs are integral to this process by adding or removing acetyl groups to/from histone or non-histone proteins, thus changing chromatin accessibility, or protein structure, function, or half-life.

Around 80–90% of proteins are acetylated at the N-terminus of the polypeptide chain during translation (Drazic et al., 2016). This N-terminal acetylation is an abundant irreversible process and is carried out by enzymes referred to as N-terminal acetyltransferases (NATs), that transfer an acetyl group from acetyl-coenzyme A to the α-amino acid of proteins. Such modifications can alter the way the proteins form their tertiary structure, affect their half-life, or even their localization within the cell (Deng and Marmorstein, 2021). While NATs can regulate the acetylation of terminals mostly during translation, post-translational acetylation, which is the main subject of this review, occurs on the ε-amino group of lysine residues in a polypeptide chain and can occur as a reversible process. Post-translational acetylation is involved in a wide variety of processes, such as maintaining cellular homeostasis, protein folding, and protein localization, and is a tightly regulated process. The enzymes involve in maintaining this homeostasis are known as histone acetyltransferases (HATs) and histone deacetylases (HDACs; Figure 1; Peserico and Simone, 2010).

## Histone acetyltransferases and histone deacetylases

### HAT enzymes

Histone acetyltransferases are ubiquitously expressed and are broadly classified based on their localization within either the nucleus or cytoplasm. Nuclear HATs are chiefly responsible for the regulation of gene expression by acetylating the nuclear histones resulting in altered chromatin compaction and subsequent gene expression. Cytoplasmic HATs acetylate proteins in the cytoplasm; this includes cytoplasmic histone proteins, which are then transported into the nucleus for further modification. HATs regulate the acetylation process by transferring an acetyl group from acetyl-coenzyme A to the ε-amino group of lysine residues (Roth et al., 2001). Depending on their functional activities, structure, and sequential homology, HATs can be further grouped into three major families; (i) the GNAT family, (ii) the MYST family, and (iii) the EP300/CREBBP family (Kamei et al., 1996; Roth et al., 2001).

The GNAT family (or KAT2/GCN5-related N-acetyltransferases) acetylates both histones and non-histone proteins (Roth et al., 2001; Kim et al., 2010; Tapias and Wang, 2017) and is responsible for a wide variety of functions including transcription regulation, response to stress, and development (Tapias and Wang, 2017; Shirmast et al., 2021). Some of this superfamily is NATs, involved in translational modification, while the remaining enzymes are involved in post-translationally acetylating lysine residues of proteins (Deng and Marmorstein, 2021). The MYST family is named from its four core enzymes which are MOZ/histone acetyltransferase 6A, Ybf2/SaS3, SaS2, and Tip60/histone acetyltransferase 5 (Kim et al., 2010). The MYST family HATs interact with many of the core nuclear histones including H2A, H2AZ, H3, and H4 (Marmorstein, 2001; Roth et al., 2001). These enzymes are highly conserved in eukaryotes and regulate essential cellular functions of gene transcription, DNA replication and damage repair, and neurogenesis (Avvakumov and Côté, 2007; Tapias and Wang, 2017). The E1A binding protein p300/Cyclic adenosine monophosphate Response Element Binding protein (EP300/CREBBP) family includes the conserved HAT enzymes p300 and CREB binding protein (CBP), which are homologous and functionally similar (Roth et al., 2001; Yang, 2004). This family also has both nuclear histone and non-histone targets (Table 1; Roth et al., 2001; Yang, 2004). CBP and p300 are involved in gene expression by recruiting transcriptional machinery through acetylation and responding to cellular hypoxia, cellular differentiation, and early brain development (Arany et al., 1996; Tapias and Wang, 2017).

**Table 1:** HAT enzymes and their roles in neurodegenerative disease (NDD).

**HAT family**	**Representative enzymes**	**Subcellular Localisation**	**Histone substrates**	**Examples of non-histone substrates**	**Identified eoles in brain**	**Identified involvement in** *NDD
GNAT family (Roth et al., 2001; Yang, 2004; Kim et al., 2010; Wang Y. et al., 2014; Wang et al., 2020)	HAT1	Nucleus/Cytoplasm	H4, H2A	HMG-N2/HMG17, AR, c-MYC, p53, and Ku70	-	-
	Gcn5	Nucleus	H2B, H3		Neural progenitor differentiation (Martínez-Cerdeño et al., 2012)	PD (Fan et al., 2020), *SCA7 (Chen et al., 2012)
	PCAF	Nucleus	H3		Regulates memories (Martínez-Cerdeño et al., 2012)	AD (Park et al., 2015), HD (Bodai et al., 2012)
	HPA2	Nucleus	H3, H4		-	-
	HPA3	Nucleus	H4		-	-
	Elp3	Nucleus/Cytoplasm	H3, H4		Dendritic and axonal branching (Xu et al., 2000; Kim C. H. et al., 2012)	ALS (Simpson et al., 2009)
Elp1	Nucleus	H3, H4		-
MYST family (Roth et al., 2001; Yang, 2004; Kim et al., 2010; Wang Y. et al., 2014; Wang et al., 2020)	TIP60	Nucleus/Cytoplasm			Embryonic neuronal development (Georgala et al., 2011; Kim C. H. et al., 2012)	AD (Müller et al., 2008), SCA1 (Gehrking et al., 2011)
	MORF	Nucleus	H2A, H3, H4		Adult neurogenesis (Thomas et al., 2000; Kueh et al., 2011)	AD (Li et al., 2021)
	MOZ	Nucleus	H3, H4		Regulation of neuronal progenitor cells & patterning genes (Katsumoto et al., 2006; Kueh et al., 2011; Perez-Campo et al., 2014; You et al., 2015)	AD (Li et al., 2021)
	MOF	Nucleus/Mitochondria	H4		AD (Li et al., 2021)
	HBO1	Nucleus	H4		AD (Li et al., 2021)
	Esa1	Nucleus	H2AZ, H4		-	-
	Sas2	Nucleus	H4		-	-
	Sas3	Nucleus	H3		-	-
p300/CREBBP (Roth et al., 2001; Yang, 2004; Kim et al., 2010; Wang Y. et al., 2014; Wang et al., 2020)	p300	Nucleus/Cytoplasm	H2A, H2B, H3, H4	HMG1, HMG-I(Y), p53, GATA-1, TCF, IF-2, ACTR (nuclear receptor coactivator), and SRC-1	Neuronal tube development (Katsumoto et al., 2006; Kueh et al., 2011; Perez-Campo et al., 2014; You et al., 2015) and memory formation (Li et al., 2002; Korzus et al., 2004)	AD (Lu et al., 2014), PD (Kontopoulos et al., 2006), HD (Bodai et al., 2012)
	CBP	Nucleus/Cytoplasm	H2A, H2B, H3, and H4	AD (Caccamo et al., 2010), PD (Kontopoulos et al., 2006; Song et al., 2010), HD (Cong et al., 2005), ALS (Rouaux et al., 2003), and *SCA3 (Chai et al., 2002)

AR, Androgen receptor; GATA-1, GATA-binding factor 1; HMG, high mobility group chromosomal protein; IF2, Initiation factor 2; SCA1, spinocerebellar ataxia type 1; SCA3, spinocerebellar ataxia type 3; SCA7, spinocerebellar ataxia type 7; SRC, Proto-oncogene tyrosine-protein kinase; and TCF, T-cell factor.

* NDD: neurodegenerative disease.

Histone acetyltransferase enzymes have many critical roles in both the developing and adult brain. For example, in adult mice, PCAF, a member of the GNAT family has been shown to have a key regulatory function in the formation of both short-term and long-term memory following stress and anxiety (Martínez-Cerdeño et al., 2012). Moreover, knockdown of either or both Elp1 and Elp3 enzymes (GNAT family), was associated with defective dendritic and axonal branching in motor neurons along with poor migration and maturation of neuronal cells (Xu et al., 2000; Kim C. H. et al., 2012). The importance of these HATs can also be seen through their roles in the developing brain, as studies have shown conditional deletion of Gcn5 (GNAT family) resulted in a 26% loss of brain mass, due to dysregulation of neural progenitor differentiation (Martínez-Cerdeño et al., 2012). The MYST family enzymes are also vital for neuronal development and adult nervous system function. For instance, overexpression of TIP60 in mouse retinas at postnatal day 4 led to an increased level of PAX6, which plays an essential role in embryonic neuronal development (Georgala et al., 2011; Kim C. H. et al., 2012). Although there are few studies examining the role of MOZ, MOF, and HBO1 in the brain, the absence of these enzymes has been shown to result in impaired regulation of neuronal progenitor cells, embryonic lethality, and reduction of neuronal patterning genes (Katsumoto et al., 2006; Kueh et al., 2011; Perez-Campo et al., 2014; You et al., 2015). Another MYST family member, MORF, is highly expressed in both the embryonic and adult brain and it has been postulated that it plays a role in adult neurogenesis (Thomas et al., 2000; Kueh et al., 2011). Both, p300 and CBP have been shown to be expressed at a high level in the neural tube of mice and are essential for neuronal tube development (Katsumoto et al., 2006; Kueh et al., 2011; Perez-Campo et al., 2014; You et al., 2015). They are also thought to be critical to homeostasis and some aspects of memory formation in the adult brain, due to high expression in young and adult mice (Li et al., 2002; Korzus et al., 2004). Moreover, studies suggest that p300 may be involved in oligodendrocyte differentiation in the developing rat brain (Zhang et al., 2016). While it is important to note the varied functions of HATs in brain function, this process does not function in isolation and is tightly regulated by balancing acetylation and deacetylation.

### HDAC enzymes

To allow regulation of the homeostasis of cellular processes controlled by lysine acetylation through HATs, the process of acetylation must be able to be reversed. This occurs through enzymes known as HDACs. HDACs were named after their initially identified role in deacetylating nuclear histone proteins to regulate transcription. However, HDACs are now recognized as playing important roles in deacetylating non-nuclear histones and non-histone proteins (Table 2) both in the nucleus and cytoplasm that have other cellular mechanisms including metabolism, protein degradation, and modulation, facilitating DNA damage repair, immune process, oxidative stress, angiogenesis, and apoptosis (Ruijter et al., 2003; Seto and Yoshida, 2014; Van Helleputte et al., 2014; Didonna and Opal, 2015; Li G. et al., 2020). Eighteen different HDACs have been identified in humans, which are known as HDAC1-11 and SIRT1-7. These are classified into four major classes named class I–IV depending on their distinguishable role in cellular processes and the subcellular regions in which they function (Glozak et al., 2005). The superfamily of HDAC proteins can be first divided into two classes depending on the type of cofactor involved in deacetylation; the zinc-dependent HDACs’ (also known as classical HDACs) and the sirtuin HDACs, which use nicotinamide adenine dinucleotide as a cofactor. The zinc-dependent HDACs are further subclassified into classes I, II, and IV whereas the sirtuin family makes up the class III HDAC enzymes depending on their functional and structural properties (Park and Kim, 2020).

**Table 2:** HDAC enzymes and their roles in neurodegenerative disease (NDD).

**HDAC classes**	**Representative enzymes**	**Subcellular Localization**	**Histone substrates**	**Examples of non-histone substrates**	**Identified roles in brain**	**Identified involvement in NDD**	**Mislocalization in NDD**
Class I (Ma and Schultz, 2008; Dovey et al., 2010; Yao and Yang, 2011; Lv et al., 2012; Wang et al., 2012; Chakrabarti et al., 2015; Park and Kim, 2020)	HDAC1	Nucleus	H4	MEF2, ATM, p53, MeCP2	Neurogenesis in developing brains (Cunliffe, 2004; Yamaguchi et al., 2005; Ye et al., 2009; Harrison et al., 2011), dendritic growth (Tea et al., 2010) & microglial maturation (Alliot et al., 1999)	AD (Pao et al., 2020), HD (Jia et al., 2012b), FTLD (Pao et al., 2020)	Cytoplasm (Simões-Pires et al., 2013)
	HDAC2	Nucleus	H2A/2B, H3,H4	NF-κB, MeCP. IRS-1	AD (Pao et al., 2020), HD (Mielcarek et al., 2011), ALS (Janssen et al., 2010)	-
	HDAC3	Nucleus	H4	NF-κB, HDAC4, HDAC5, HDAC7, HDAC9, pRb	Regulating neuronal homeostasis (Soriano and Hardingham, 2011; Norwood et al., 2014), axonal regeneration & memory formation (Fischle et al., 2002; McQuown et al., 2011)	AD (Pao et al., 2020), ALS (Janssen et al., 2010), HD (Jia et al., 2012b)	-
	HDAC8	Nucleus/Cytoplasm	H2A/2B	HSP70	Neuronal differentiation (Katayama et al., 2018) & skull development (Haberland et al., 2009a)	PD (Quan et al., 2021)	-
Class IIa (Yao and Yang, 2011; Wang et al., 2012; Wang Z. et al., 2014; Caslini et al., 2019; Park and Kim, 2020; Wang et al., 2021)	HDAC4	Nucleus/Cytoplasm	H4	FOXO, p53, p21, HDAC3, Runx2, MEF2	Memory formation and synaptic plasticity (Broide et al., 2007; Kim M. S. et al., 2012), Purkinje cell differentiation & postnatal cerebellum development (Majdzadeh et al., 2008)	AD (Chen Y.-A. et al., 2020), PD (Wu et al., 2017), HD (Mielcarek et al., 2011), ALS (Pegoraro et al., 2020), FTLD (Whitehouse et al., 2015)	Cytoplasm (Mielcarek et al., 2013)/Nucleus (Wu et al., 2017)
	HDAC5	Nucleus/Cytoplasm	H4	HDAC3, MEF2, Runx2	Differentiation of neuronal stem cells (Schneider et al., 2008)	AD (Agis-Balboa et al., 2013), PD (Mazzocchi, 2021), HD (Yeh et al., 2013), ALS (Janssen et al., 2010), FTLD (Whitehouse et al., 2015)	-
	HDAC7	Nucleus/Cytoplasm	H3	HDAC3, PML, Runx2, MEF2	Dendric growth, neurogenesis, neuronal protection, & maturation (Lai et al., 2010; Sugo et al., 2010; Ma and D’Mello, 2011)	AD (Lu et al., 2019), PD (Mazzocchi, 2021)	-
	HDAC9	Nucleus/Cytoplasm	H3, H4	HDAC3, MEF2, CAM	-
Class IIb (Yao and Yang, 2011; Wang et al., 2012; Park and Kim, 2020)	HDAC6	Cytoplasm	-	α-tubulin, Cortractin, HDAC11, HSP90, PP1	Dendritic growth & branching (Kim et al., 2009)	AD (Simões-Pires et al., 2013), PD (Simões-Pires et al., 2013), HD (Simões-Pires et al., 2013), ALS-FTLD (Simões-Pires et al., 2013), CMT (d'Ydewalle et al., 2011)	Perinuclear (Simões-Pires et al., 2013)
	HDAC10	Cytoplasm	-	LcoR, and PP1	Dendric growth, neurogenesis, neuronal protection, and maturation (Lai et al., 2010; Sugo et al., 2010; Ma and D’Mello, 2011)		-
Class III (Yao and Yang, 2011; Wang et al., 2012; Seto and Yoshida, 2014; Park and Kim, 2020)	SIRT1	Nucleus/Cytoplasm	H3, H4	NF-κB, p300, p53, Ku70, PCAF, TIP60	Neuronal development, axonal growth, regeneration, & neuroprotection (Pandithage et al., 2008; Tiberi et al., 2012; Li X. H. et al., 2013; Kaluski et al., 2017; Sidorova-Darmos et al., 2018; Garcia-Venzor and Toiber, 2021)	AD (Bonda et al., 2011), PD (Li X. et al., 2020), HD (Jęśko et al., 2017), ALS (Outeiro et al., 2008)	Cytoplasm (Zschoernig and Mahlknecht, 2008)
	SIRT2	Cytoplasm	H3, H4	α-tubulin	AD (Jęśko et al., 2017), PD (Outeiro et al., 2007), HD (Outeiro et al., 2007)	-
	SIRT3	Mitochondria	H4	Ku70, GDH, SOD2	AD (Jęśko et al., 2017), PD (Salvatori et al., 2017), HD (Salvatori et al., 2017), ALS (Salvatori et al., 2017)	-
	SIRT4	Mitochondria	-	GDH	AD (Jęśko et al., 2017), ALS (Körner et al., 2013)	-
	SIRT5	Mitochondria	-	Cytochrome c, CPS1	AD (Akter et al., 2021) PD (Maszlag-Török et al., 2021), ALS (Harlan et al., 2019)	-
	SIRT6	Nucleus	H3	TNF-α	ALS (Körner et al., 2013)	-
	SIRT7	Nucleus	H3	p53	-	-
Class IV (Wang et al., 2012; Núñez-Álvarez and Suelves, 2022)	HDAC11	Nucleus/Cytoplasm	H3, H4	HDAC6, Cdt1	Maturation and development of neuronal cells and oligodendrocytes (Liu et al., 2008, 2009)	HD (Kumar et al., 2022), ALS (Janssen et al., 2010)	-

ATM, Ataxia-telangiectasia-mutated; CAM, calmodulin; CPS1, Carbamoyl phosphate synthetase 1; Cdt1, chromatin licensing and DNA replication factor 1; FOXO, Forkhead box O; GDH, glutamate dehydrogenase; HSP, heat shock protein; IRS, insulin receptor substrate; LcoR, ligand-dependent receptor co-repressor; MeCP, methyl-CpG-binding domain protein; MEF, myocyte enhancer factor; NF-κB, nuclear transcription factor-kappa B; PP1, protein phosphatase, pRb, retinoblastoma protein; Runx, Runt-related transcription factor; SOD, superoxide dismutase; and TNF, tumor necrosis factor.

The class I HDACs includes HDAC1, 2, 3, and 8, and while most function within the nucleus, HDAC3 shuttles between the nucleus and cytoplasm for transcriptional regulation (Yao and Yang, 2011; Didonna and Opal, 2015; Park and Kim, 2020). Class II HDACs are further classified into class IIa and IIb based on their structural specifications. HDAC4, 5, 7, and 9 all contain a common N-terminal binding domain and belong to the class IIa HDACs, while HDACs 6 and 10 belong to the class IIb group. Class IIa HDACs shuttle between the nucleus and cytoplasm whereas class IIb enzymes are predominantly localized to the cytoplasm (Marsoni et al., 2008; Li G. et al., 2020). The class III HDACs (SIRT1-SIRT7) are structurally distinct from class I and class II HDACs. Among the SIRT proteins, SIRT1, 6, and 7 are abundant in the nucleus, although under certain conditions SIRT1 can be retained in the cytoplasm (Frye, 2000; Blander and Guarente, 2004; Michishita et al., 2005; Ramadori et al., 2008; Shoba et al., 2009). On the other hand, SIRT3, 4, and 5 are primarily present in mitochondria and SIRT2 is commonly found in the cytoplasm (Frye, 2000; Blander and Guarente, 2004; Michishita et al., 2005; Ramadori et al., 2008; Shoba et al., 2009). Lastly, class IV, HDACs contain only HDAC11. This enzyme carries homologous features to class I and II HDACS. HDAC11 is mainly found in the nucleus; however, it can be found co-localized with HDAC6 in the cytoplasm (Ruijter et al., 2003; Seto and Yoshida, 2014).

The function and localization of HDACs have been investigated in several different models. For example, class I HDAC enzymes are ubiquitously expressed at different levels in all tissues (Uhlén et al., 2015), whereas the other classes HDACs may have more tissue-specific expression (Kuwahara et al., 2003; Nakagawa et al., 2006; Zupkovitz et al., 2006; Montgomery et al., 2007; Knutson et al., 2008; Trivedi et al., 2008; Soriano and Hardingham, 2011). Multiple studies have shown the critical roles of these enzymes in developing the heart, skeletal muscle, liver, bone, vascular system, and immune system (Kuwahara et al., 2003; Chang et al., 2004; Méjat et al., 2005; Chang et al., 2006; Cohen, 2006; Nakagawa et al., 2006; Zupkovitz et al., 2006; Arnold et al., 2007; Montgomery et al., 2007; Knutson et al., 2008; Trivedi et al., 2008; Soriano and Hardingham, 2011; Seto and Yoshida, 2014; Parra, 2015; Li et al., 2016; Wang et al., 2017; Liu et al., 2020). All these HDACs are expressed in the brain and the class IV HDAC11 is expressed at the highest level in the brain compared to other classes (Frye, 2000; Blander and Guarente, 2004; Michishita et al., 2005; Broide et al., 2007; Ramadori et al., 2008; Haberland et al., 2009b; Shoba et al., 2009; Parra, 2015; Uhlén et al., 2015; Wey et al., 2016).

Like HATS, HDACs have been shown to be responsible for carrying out essential functions in both the developing and adult brains, such as maintaining synaptic plasticity, dendritic outgrowth, and axon regeneration (Morris and Monteggia, 2013). Class 1 HDACs may have a particularly important role in cortical development and function (Morris and Monteggia, 2013), due to their high expression in cortical tissue (Broide et al., 2007; Wey et al., 2016). Furthermore, studies have shown that both HDAC1 and HDAC2 have roles in neurogenesis in developing brains (Cunliffe, 2004; Yamaguchi et al., 2005; Ye et al., 2009; Harrison et al., 2011) as their deletion in glial fibrillary acidic protein (GFAP)-Cre transgenic mice led to an impairment in the generation of neurons from neural progenitors and increased neuronal death (Montgomery et al., 2009). Studies have also demonstrated that HDAC2 is involved in memory formation and regulating synaptic plasticity by restricting the maturation of adult neuronal synapses (Guan et al., 2009). Studies in the olfactory system of *Drosophila* suggest that both HDAC1 and HDAC2 are involved in the establishment of nervous system connections as they are required for appropriate dendritic growth (Tea et al., 2010) along with facilitating microglial maturation and function (Alliot et al., 1999). HDAC3, which is expressed at the highest level in the rat brain compared to other enzymes from class I (Broide et al., 2007) is responsible for regulating neuronal homeostasis (Soriano and Hardingham, 2011; Norwood et al., 2014). Studies in transgenic mice suggest that HDAC3 may also play a role in axon regeneration and negatively regulate long-term memory formation (Fischle et al., 2002; McQuown et al., 2011). Little is known about the regulatory roles of HDAC8 in brain function, but it is the least expressed from its class in the rat brain (Broide et al., 2007). However, it has been shown to negatively regulate neuronal differentiation (Katayama et al., 2018), and it regulates skull development in vertebrates, where the absence of this enzyme caused skull instability followed by perinatal lethality in a mouse model (Haberland et al., 2009b).

Although studies have revealed vital roles of class II HDACs in CNS development, most of the functions of these enzymes are still unknown. HDAC4 has been shown to impair memory formation and synaptic plasticity when knocked out of mice (Broide et al., 2007; Kim M. S. et al., 2012) and is essential for regulating postnatal cerebellum development and Purkinje cell differentiation in posterior lobes (Majdzadeh et al., 2008). However, Price and colleagues (2012) did not see abnormalities when HDAC4 was knocked out of forebrain neurons (Price et al., 2013) suggesting cell/region-specific roles for HDAC4 during early CNS development. HDAC5, the second most highly expressed class II enzyme in brain tissue, has been found to be involved in facilitating the differentiation of neuronal stem cells (Schneider et al., 2008). Several HDACs have roles in the cytoplasm of neurons and are involved in axonal integrity and dynamics. For example, HDAC5, much like HDAC3 and HDAC6, may be involved in axonal regeneration following injury by deacetylating microtubules, which is required for optimizing growth cone dynamics (Cho and Cavalli, 2012). Studies in cerebral and hippocampal cells have shown that HDAC6 like HDAC2 plays a crucial role in regulating the processes of dendritic growth and branching (Kim et al., 2009). Although HDAC6 was upregulated during axonal injury and neuronal oxidative stress (Witte et al., 2008), its role in development may be less important, as HDAC6 knockout mice have not shown any abnormalities other than increased microtubule acetylation (Zhang et al., 2008). The other HDACs from this class, HDAC7, 9, and 10, have also been shown to function in dendric growth, neurogenesis, neuronal protection, and maturation (Lai et al., 2010; Sugo et al., 2010; Ma and D'Mello, 2011). The class IV HDAC11 is yet to be studied widely, however, a few studies have demonstrated its function in the maturation and development of neuronal cells and oligodendrocytes (Liu et al., 2008, 2009).

Among the class III sirtuins, SIRT2, SIRT3, and SIRT5 are most abundantly expressed in the brain (Sidorova-Darmos et al., 2014) and enzymes from this class are also involved in regulating neuronal development, axonal growth, regeneration, and neuroprotection like other HDACs (Pandithage et al., 2008; Tiberi et al., 2012; Li X. H. et al., 2013; Kaluski et al., 2017; Sidorova-Darmos et al., 2018; Garcia-Venzor and Toiber, 2021). However, genetic modifications of most of these HDACs in animal models have been shown to be embryonically lethal, as such their core roles are remained to be elucidated.

## Role of altered acetylation in neurodegenerative disease

A number of different PTMs have been implicated in the pathogenesis of neurodegenerative diseases (Didonna and Benetti, 2016). Most studies have focused on examining alterations to phosphorylation (Tenreiro et al., 2014); acetylation has been less well studied. However, dysregulation of acetylation has been reported to occur in several neurodegenerative diseases, such as AD, ALS, and HD (Saha and Pahan, 2006) and although not well characterized, some studies have postulated that dysregulation of HATs and HDACs may be paramount to the onset and/or progression of neurodegenerative disease (Saha and Pahan, 2006). For example, HATs and HDACs interact with a range of non-histone substrates that may be implicated in neurodegenerative diseases, such as p53, NF-KB, and STAT1 (Boutillier et al., 2003; Rouaux et al., 2003), while failing to maintain appropriate regulation of HATs and HDACs can result in activation of apoptotic pathways and widespread dysregulation in neuronal cells (Rouaux et al., 2003). Whether alterations to these enzymes are upstream drivers of neurodegeneration or secondary to other pathological processes is not well understood. Janssen et al. (2010) demonstrated that increased levels of HDAC2 and reduced HDAC11 mRNA were related to apoptotic neuronal death in human brain tissue from people diagnosed with ALS (Janssen et al., 2010). Additionally, accumulated mutant huntingtin (htt) protein in HD has also been shown to interact with the HAT domain of the CBP enzyme, decreasing HAT activity in post-mortem human brain tissue (Rouaux et al., 2004).

There is much work to do to fully understand alterations to the enzymes involved in acetylation as well as alteration to acetylation of proteins associated with neurodegenerative disease. Here we review the current literature on two key pathways implicated in neurodegenerative diseases; proteostasis (protein folding, aggregation, and degradation) as well as correct functioning of the cytoskeleton. We review both the role of acetylation in these physiological processes as well as reported alterations in disease since it is important to know how these physiological processes are regulated through acetylation to understand the dysregulations in neurodegenerative diseases.

### Protein folding, aggregation, degradation, and metabolism

The prerequisite step for a functionally active protein is its folding into a three-dimensional structure. This protein folding process occurs in the endoplasmic reticulum (ER) and involves PTMs including acetylation (Stevens and Argon, 1999). Acetylation of proteins is also important for controlling their degradation. Each intracellular protein or protein complex has a specific lifespan after which it is degraded by the proteasome or by autophagy and acetylation has a crucial role in regulating both. For example, it has been shown that acetylated proteins are protected from ubiquitin-induced protein modification and degradation by the proteasome which increases the lifespan and functions of these proteins (Knorre et al., 2009). Conversely, studies have shown that histone degradation requires the addition of acetyl groups to undergo the proteasome activator PA200/Blm10-based degradation process (Qian et al., 2013). Furthermore, acetylation also controls aspects of the degradation machinery. Acetylation can aid in the regulation of autophagy from activation of core autophagic proteins, fusion of autophagosomes with lysosomes, and autophagic cargo assembly (Xu and Wan, 2022). ATG proteins, which play an essential role in phagophore formation, are maintained by HATs/HDACs to fine-tune the inhibition or activation of autophagy (Bánréti et al., 2013). For example, acetylation of ATG9A autophagic proteins in the lumen of the ER prevents activation of autophagosomes whereas deacetylation of ATG9A induces the formation of autophagosomes (Pehar and Puglielli, 2013). During stress conditions, like starvation, SIRT1 deacetylates these ATG proteins to facilitate autophagy (Bánréti et al., 2013). Additionally, acetylation of microtubules also occurs in response to stress and activates the MAPK/JNK autophagic signaling pathway (Bánréti et al., 2013). Alongside the established roles of acetylation as a post-translational modification, it is also linked to many aspects of metabolism. Studies have demonstrated that the acetyl-CoA metabolite availability is essential for acetylation to occur during histone or non-histone protein modifications (Peleg et al., 2016). This largely depends on the extent of mitochondrial production of acetyl-CoA available within the cell (Peleg et al., 2016). Additionally, other metabolite cofactors such as NAD^+^ are required for the deacetylase activities of sirtuins (Choudhary et al., 2014). While mitochondrial and metabolic activity is essential for acetylation to occur, studies have also shown that acetylation can regulate mitochondrial homeostasis, including storage and utilization of cellular energy. For example, a deficit of SIRT3 results in the production of reactive oxygen species along with altered oxidative metabolism (Guarente, 2011; Baeza et al., 2016). Interestingly, most of the enzymes involved in metabolic processes such as glycolysis, urea cycle, and gluconeogenesis have been found to be acetylated (Arif et al., 2010).

### Acetylation and altered proteostasis in neurodegenerative disease

In neurodegenerative diseases, altered acetylation has been implicated in contributing to failure of protein clearance mechanisms through autophagy and the proteosome (Sambataro and Pennuto, 2017; Son et al., 2021). For instance, studies have reported that increased activity of p300/CBP enzyme altered autophagic flux resulting in excessive secretion of tau protein in transgenic AD mice (Chen X. et al., 2020). Furthermore, the HDAC inhibitor, 4b (preferentially inhibit HDAC1 and HDAC3 enzymes), was shown to improve cognitive function in a transgenic HD mouse model by clearing Huntingtin protein through proteasome and lysosome pathways (Jia et al., 2012a). Altered proteostasis may also result from protein aggregation, which is a pathophysiological hallmark for neurodegenerative diseases (Tutar et al., 2013). Aggregation is thought to result from several different conditions occurring in aggregate-prone proteins such as mutations, oxidative stress, or altered PTM causing proteins to misfold and generate insoluble aggregates (Tutar et al., 2013). These aggregates are thought to impair the structural and functional activities of neurons, which further facilitate the pathogenic process of disease conditions in AD, PD, FTLD, HD, and ALS (Tutar et al., 2013).

The majority of research investigating links between protein aggregation and PTMs has focused on abnormal phosphorylation, however, as most of these disease-related proteins can become acetylated, alterations to acetylation could contribute to protein misfolding and protein aggregate formation in neurodegenerative diseases (Schaffert and Carter, 2020). Here we focus on what we know about altered acetylation in some of the key proteins which become aggregated in neurodegenerative diseases.

#### Transactive response DNA binding protein 43 in ALS/FTD

Transactive response DNA binding protein 43, or TDP-43 is a transcription and RNA metabolism regulator protein primarily localized to the nucleus (Sephton et al., 2010). This protein is encoded by the human *TARDBP* gene and contains two RNA recognition motifs, a prion-like domain in the C-terminal region and a folded N-terminal domain. Like many proteins, TDP-43 also undergoes PTMs (François-Moutal et al., 2019). Protein pathology has been linked to hyperphosphorylation of the protein; however, more recent studies have implicated acetylation in the normal and pathological role of TDP-43. It has been shown that CBP-associated acetylation of lysine 145 and 192 regulates the binding of TDP-43 with target RNA and is a core site for regulating additional acetylation events (Cohen et al., 2015; Buratti, 2018). Another study demonstrated that the regulatory role of acetylation at lysine 136 site of TDP-43 where transfection with SIRT1 targeting lysine 136 sites reduced TDP-43 aggregation in studied sh^TDP-43^-HEK293E cells (Garcia Morato et al., 2022). In ALS/FTD (frontotemporal dementia), studies have shown that hyperacetylation of TDP-43 caused a reduction in its splicing ability to targeted RNA and led to the aggregation of TDP-43 in the cytoplasm, inducing neuronal stress (Buratti, 2018). Acetylation of lysine 82 and lysine 192 of TDP-43 has also been associated with pathogenic mislocalization of TDP-43 to the cytoplasm (Kametani et al., 2016).

#### Fused in sarcoma in ALS/FTD

Fused in sarcoma (FUS), like TDP-43, is a predominantly nuclear RNA binding protein involved in RNA metabolism. This protein has been shown to contain N- and C-terminal nuclear localization signal (NLS) sites which undergo acetylation to regulate their function (Bock et al., 2021; Farina et al., 2021). Furthermore, acetylation at lysine 315/316 in the RNA recognition motif of FUS, regulates its binding with RNA whereas acetylation at lysine 510 of the C-terminal NLS may facilitate increased aggregation of the protein in the cytoplasm (Farina et al., 2021). Moreover, a liquid chromatography assay revealed that imbalance of N-terminal acetylation may also prompt FUS to aggregate in ALS/FTD diseases (Bock et al., 2021; Farina et al., 2021).

#### Tau (MAPT) in AD and PD

Tauopathy is one of the major pathophysiological signatures of both AD and FTLD. Tau is a microtubule-associated protein (MAP) that has a crucial role in assembling and stabilizing microtubules (Weingarten et al., 1975; Goedert et al., 1989). Phosphorylation of tau and its links to neurodegenerative diseases have been extensively studied (Brotzakis et al., 2021), however acetylation of tau also occurs at the N-terminus and lysine residues and may regulate the binding of tau with microtubules (Derisbourg et al., 2015; Brotzakis et al., 2021). For example, in post-mortem AD tissue as well as in transgenic mouse model of tauopathy, the amino acid KXGS motif, which resides in the microtubule-binding region of tau protein was hypoacetylated which was shown to impair tau activity and result in accumulation in neurofibrillary tangles (Cook et al., 2014a). Additionally, hyperphosphorylation of this motif prevents tau acetylation which again causes dissociation of tau from microtubules, reducing the stability of microtubules and axonal transport (Cook et al., 2014b). While hypoacetylation of tau has been associated with increased toxicity, research also points to a relationship between elevated acetylated tau and tauopathies in AD (Min et al., 2010). A study by Sohn et al. (2016) demonstrated that hyperacetylation of tau was involved in the pathogenesis of AD, by impairing axonal initial segment and microtubule dynamics. The axon initial segment functions to maintain neuronal polarity between the axonal domain and somatic dendritic domain. Sohn demonstrated an increased acetylated level of tau at lysines 274 and 281 and destabilization of the axon initial segment in the superior temporal gyrus of human AD brain tissue (Sohn et al., 2016). In the human neuroblastoma-derived SHSY5Y PD model, both tau and α-synuclein, a key pathological PD protein, were found to be hyperacetylated, which was linked to HAT p300 modulation of the deacetylase enzymes SIRT2 and HDAC6 (Esteves et al., 2019). Beyond functional modifications related to altered tau acetylation itself in neurodegenerative disease, altered acetylated tau has been related to neurofibrillary tangle formation in AD through reduced clearance of tau protein. It has been shown that acetylation at lysine 311 of the human leukocyte antigen DRB1*04, which specifically binds with tau, is associated with the clearance of tau by T cells, and subsequent slowing down of neurodegeneration (Le Guen et al., 2021). Altered acetylation of tau has also been associated with neurodegenerative diseases like Pick’s disease and corticobasal degeneration in human tissue. It was reported that insoluble and aggregated neurofibrillary tangles in human tissue from these diseases had altered the acetylation of the K280 lysine residues of tau (Cohen et al., 2011). Additionally, there are other examples where acetylation of microtubules has altered binding of molecules such as of motor proteins dynein/dynactin and kinesin-1 to microtubules, or alter axonal transport such as of BDNF in HD (Dompierre et al., 2007). Acetylation of microtubules may further facilitate binding of microtubule-associated proteins such as tau, which can interact with tubulin and alter the pathophysiological events of accumulated tau proteins in neurodegenerative diseases (Selenica et al., 2014; Saunders et al., 2022).

#### Alpha-synuclein in PD and dementia with Lewy bodies

Alpha-synuclein aggregates are core pathology in PD and dementia with Lewy bodies. Under normal conditions, this protein is predominantly found in neuronal presynaptic terminals and is involved in regulating synaptic vesicle trafficking for the release of neurotransmitters (Vargas et al., 2014). Alpha-synuclein undergoes N-terminal acetylation which modulates its binding with tubulin, actin, and lipids (Iyer et al., 2016; Deng et al., 2020). Additionally, in studies of PD, altered acetylation of α-synuclein at lysine 6 and 10 resulted in aggregation leading to synucleinopathies and neuronal toxicity (De Oliveira et al., 2017; Vinueza-Gavilanes et al., 2020).

#### Huntingtin in Huntington’s disease

The function of htt protein which is aggregated in Huntington’s disease is not well understood, however, it has been implicated in axonal transport and vesicle trafficking (DiFiglia et al., 1995; Vitet et al., 2020). Mass spectroscopy of the protein synthesized in HEK 293 T cell lines demonstrated five acetylation sites including acetylation at lysine 9, 178, 236, 345, and 444 (Cong et al., 2011). These acetylation sites along with other PTMs have been implicated in the physiological and pathological functions of huntingtin protein, such as modifying its structure, oligomerization, modulating the binding with membrane and other proteins, and formation of fibrils (Chiki et al., 2017). Further research has shown that huntingtin binding protein, HYPK, is involved in regulating N-terminal acetylation, while mutation of HYPK in HD reduces the aggregation of huntingtin protein through altered N-terminal acetylation (Arnesen et al., 2010; Gottlieb et al., 2021). In addition, acetylation of lysine 444 promotes the removal of aggregated huntingtin protein through autophagy which is altered in HD pathophysiology (Jeong et al., 2009).

#### Beta-amyloid in Alzheimer’s disease

The beta-amyloid (Aβ) peptide, which is a cleavage product of the amyloid precursor protein, is the core component of amyloid plaques in AD. It contains two key acetylation sites; lysine 16 and 28. Acetylation at these sites has been implicated in reducing aggregation, oligomerization, and fibril formation of Aβ peptides (Pilkington et al., 2019; Guha and Subramaniyam, 2021). In contrast, another study suggested acetylated Aβ created amorphous aggregates resulting in increased reactive oxygen species and cytotoxicity in studied SH-SY5Y neuronal cells (Adhikari et al., 2020).

#### Superoxide dismutase 1 in ALS

The reactive oxygen species scavenging enzyme superoxide dismutase 1 (SOD1) is a key pathological protein linked to neurodegeneration in ALS (Ralph et al., 2005; Barber et al., 2006). Studies have suggested acetylation of lysine 70, which is regulated by SIRT1, caused the inactivation of the antioxidant function of SOD1 (Banks and Andersen, 2019). Furthermore, acetylation at lysine site 122 suppressed mitochondrial respiration which subsequently increased the oxidative stress scavenging activity of SOD1 (Banks and Andersen, 2019). Acetylation at lysine 123 of SOD1 has been shown to have regional and cell type specificity in the healthy adult mouse CNS which may indicate its essential function in these cells (Kaliszewski et al., 2016). One study suggested alteration of acetylation at lysine 123 facilitated protein aggregation and pathogenesis in a SOD1 knockout ALS model. Not only was acetylation of lysine 123 increased, but high levels of acetylated SOD1 misfolded protein were found to be aggregated in primary cilia of astrocytes and in the vesicles which were derived from these primary cilia (Kaliszewski, 2016). These vesicles containing misfolded acetylated SOD1 were taken up by neurons, leading to neurodegeneration (Kaliszewski, 2016).

#### Clinical acetylation sites in neurodegenerative disease

Despite much research into the role of acetylation in NDD, there have been few studies to investigate sites of clinical relevance to limit neurotoxic proteins (Min et al., 2015, 2018; Dave et al., 2021). Of note, hyperacetylation of tau at lysine 174 was an early occurrence in post-mortem human AD brains and in the hippocampus of PS19 AD mice (Min et al., 2015). Acetylation at this site slowed tau turn over, promoted accumulation of the protein, and resulted in reduced hippocampal volume *in vivo*. Moreover, a model of tau lys174 deacetylation led to improved cognitive performance in behavioral tasks. Small molecule inhibition of p300 also improved cognitive outcomes in PS19 AD mice (Min et al., 2015).

## The cytoskeleton

Cytoskeletal alterations have been implicated in many neurodegenerative diseases (Cairns et al., 2004) and acetylation of many cytoskeletal proteins plays a key role in their regulation (Scott et al., 2012; Latario et al., 2020). An understanding of the normal role of acetylation in the regulation of the cytoskeleton is important to understand changes in neurodegenerative disease.

### Regulation of actin filaments

Actin filaments are involved in numerous cellular processes including maintaining cell shape and mobility and are important in neurons for neurite outgrowth, maintaining the structure of the axon as well as cytoplasmic transport (Kirkpatrick and Brady, 1999). To regulate these cellular activities, all three types of actin isoforms (alpha, beta, and gamma) have been reported to be acetylated along with actin regulatory proteins such as the actin-related protein (Arp) 2/3 complex involved in the regulation of actin filaments, and cortactin, which recruits the Arp 2/3 complex to the cortical actin cytoskeleton (Choudhary et al., 2009).

Although the impact of acetylation on these proteins is yet to be fully understood, some functional information has been demonstrated. For instance, acetylation at lysine 61 of the gamma isoform relates to the stabilization of stress fiber (Kim et al., 2006; Choudhary et al., 2009) and six subunits of the Arp2/3 complex have been shown to be acetylated to regulate actin nucleation, the first step in polymerization to F-actin (Choudhary et al., 2009), a critical component of structures such as dendritic filopodia and synapses. The F-actin regulatory protein cortactin has also been reported to be acetylated through p300/CBP (Weaver, 2008; Zhang et al., 2009) and deacetylated *via* HDAC6 and SIRT2 (Li Y. et al., 2013; Kim et al., 2020). Overexpression of HDAC6 also hinders the association of cortactin with F-actin, leading to lower levels of polymerized and branched actin (Zhang et al., 2007). Additionally, altered acetylation of cortactin by HDAC6 is involved in the fusion of autophagosomes and lysosomes, alterations to which contribute to neurodegeneration (Li Y. et al., 2013; Kim et al., 2020).

### Regulation of microtubules

**Figure 2:**
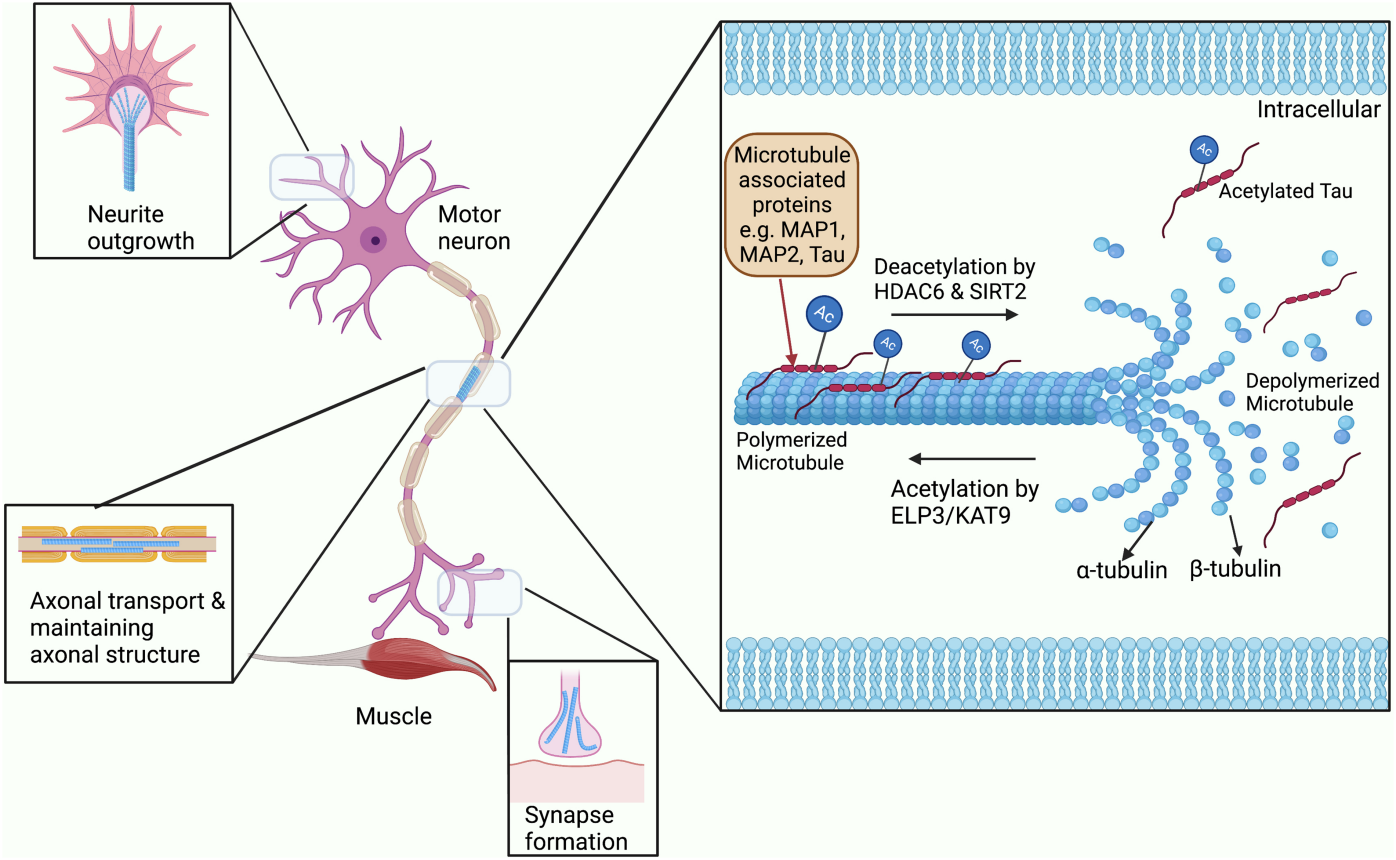
The role of acetylation and deacetylation on microtubule stability. Microtubules are constantly being modified through the addition and removal of acetylation by HATs and HDACs. Binding of Microtubule associated proteins or MAPs to microtubules is also regulated by acetylation and also affects their stability (Cohen et al., 2011). These modifications allow for the rapid expansion and removal of microtubule structures for neurite outgrowth, synapse formation, transport through the axon, and maintaining the overall structure of the axon.

Acetylation of the cytoskeleton has been most widely studied in microtubules, and interestingly the first ever cytoplasmic acetylation protein that was studied was in association with microtubules (Sadoul et al., 2010). Microtubules are hollow tubular structures that act as tracks for cargo to move down the axon as required. These hollow tubes consist of two heterodimer subunits; α- and β-tubulin, and undergo continuous structural modification allowing growth and shrinkage according to the cellular demand (Figure 2; Guo and Van Den Bosch, 2018). In addition, microtubules are found in specialized structures such as mitotic spindles which are required for cell division. These functional activities of microtubules are widely regulated by PTM processes, particularly through acetylation of their α subunit (Janke, 2014). Acetylation of microtubules has mostly been studied at the lysine residue 40 of α-tubulin, although it also occurs at other lysine residues of both α- and β-tubulin (Choudhary et al., 2009). Acetylation of tubulin is related to the formation and stabilization of microtubule bundles and increasing the level of microtubule polymerization protein TPPP/p25 (Ogawa-Goto et al., 2007; Tőkési et al., 2010). These acetylated microtubule bundles are not only found in the cytoplasm of major cellular types like neurons, but also in the microtubular substructures such as flagella, mitotic spindles, and cilia (Reed et al., 2006; Sadoul et al., 2010). In addition, acetylation aids the binding and mobility of the axonal motor proteins kinesin and dynein to the microtubules during axonal transport (Reed et al., 2006; Sadoul et al., 2010).

As mentioned above, HDAC6 plays an essential role in regulating the acetylation of both the α and β-tubulins of microtubules. Both *in vivo* and *in vitro* studies have shown that hypoacetylation or hyperacetylation of α-tubulin occurs by overexpressing or inhibiting HDAC6, respectively (Zhang et al., 2003). HDAC6 is present in the perinuclear region and is co-localized with microtubule-associated motor complexes, particularly with dynactin p150*^glued^* suggesting a role in microtubule-associated transport (Hubbert et al., 2002). Osseni et al. (2020) demonstrated that HDAC6 function is critical for neuromuscular junction stability and organization, involving not only the structure of microtubules but also the maintenance of the acetylcholine receptor clusters (Osseni et al., 2020). The class III HDAC SIRT2 also predominantly localizes to the cytoplasm and is involved in deacetylating α-tubulin at lysine-40. SIRT2 and HDAC6, when overexpressed, have been shown to coimmunoprecipitate suggesting that they belong to the same multiprotein network. However, they can be inhibited independently to achieve the hyperacetylation of α-tubulin (North et al., 2003).

### Regulation of intermediate filaments

The acetylation of intermediate filaments has been less well studied, however, both vimentin and cytokeratin 8 have been shown to be acetylated in their lysine residues destabilizing the polymer structure and affecting their ability to maintain the cellular shape and rigidity (Leech et al., 2008; Drake et al., 2009). Less is known about the acetylation of intermediate filaments expressed in the brain such as the neurofilament proteins, and astrocytes expressing GFAP (Gao et al., 2007). A study examining the effect of two global HDACs inhibitors, Trichostatin A, and Sodium Butyrate on primary cultured astrocytes demonstrated that HDACs are responsible for maintaining the ratio of the two isoforms of GFAP; GFAPδ, and GFAPα (Gao et al., 2007), through transcription and splicing. Increased levels of GFAPδ resulted in a collapse of the GFAP cytoskeletal network (Kanski et al., 2014).

### Altered acetylation of cytoskeletal proteins in neurodegenerative diseases

There is growing evidence of the links between altered cytoskeletal proteins and neurodegenerative disease, including alterations to microtubule stability which can be detrimental to axonal transport, as well as impairments in actin dynamics potentially leading to altered plasticity and accumulation of intermediate filament proteins (Luo, 2002; Saha and Pahan, 2006; Kapitein and Hoogenraad, 2015; Esteves et al., 2019). While there has been a focus on examining alterations to phosphorylation, a growing body of evidence has also implicated altered acetylation of these proteins in disease (Zhang and Benson, 2001; Cartelli et al., 2010; Esteves et al., 2019; Kim et al., 2020). The acetylation of the microtubule-associated protein tau has been discussed in the previous section; below we expand on other cytoskeletal proteins that may be implicated as contributing to neurodegenerative disease.

### Altered acetylation of microtubules

Several studies have demonstrated altered acetylation of microtubules in neurodegenerative diseases and this has been linked to facilitating the aggregation of toxic proteins in axons (Liu et al., 2012; Brunden et al., 2017). Kim C. H. et al. (2012) reported a decrease in acetylated α-tubulin in the frontal cortex of 13-month-old 5XFAD AD mice, which overproduced Aβ proteins. The decreased acetylated α-tubulin was linked to alterations in axonal transport. Multiple studies have shown that increased acetylation of microtubules can rescue axonal transport in AD mouse models (Kim C. et al., 2012; Brunden et al., 2017). Inhibiting deacetylation in an AD mouse model restored axonal transport of mitochondria, which was associated with improved cognition (Kim C. et al., 2012). Another study by Zhang et al. (2014) used the APP^swe^/PS1^ΔE9^ mouse model of AD and showed that HDAC6 was increased in this model leading to higher levels of microtubule instability. Treatment with HDAC6 inhibitors (Tubastatin A & ACY-1215) resulted in increased acetylation of α-tubulin and improvement of mitochondrial transport due to recruitment of more kinesin-1 and dynein to the microtubules, which facilitated fusion of lysosomes and autophagosomes enhancing the removal of Aβ plaques from neuronal cells. This study suggested the connection between impaired cognitive function in AD and altered acetylation of microtubules (Zhang et al., 2014). Further studies support this hypothesis, as decreased microtubule acetylation in AD has also been associated with increased microtubule severing by the severing protein katanin, destabilizing the microtubules and affecting the transport of axonal cargo (Mao et al., 2017).

In PD, a number of proteins implicated in disease, including parkin (a ubiquitin-protein ligase protein), TPPP/p25 (involved in microtubule bundle formation), PINK_1_ (autophagy triggering protein), LRRK2 (regulates immune response), and α-synuclein, are linked to microtubules and have been shown to be involved in altered microtubule acetylation and PD pathogenesis (Bonifati, 2014; Oláh et al., 2017). Esteves et al. (2019) demonstrated that there was decreased microtubule acetylation in a cybrid cell model involving PD patient mitochondrial DNA that was linked to impaired axonal transport and altered cellular distribution of mitochondria (Esteves et al., 2014). Conversely, mutant α-synuclein has been shown to hyperacetylate microtubules, which accumulated in dopaminergic neurons and result in altered binding to kinesin 1 and destabilization (Smith and Stillman, 1991; Alim et al., 2004). Several studies have linked altered acetylation of microtubules with altered interaction between LRRK2 with microtubules in growth cones. As LLRK2 is involved in stabilizing microtubule dynamics, genetic mutations to LLRK2 impact the homeostasis of microtubule acetylation and axonal transport system leading to motor impairment (Esteves and Cardoso, 2017). In addition, parkin and α-synuclein have also been linked to increased accumulation of acetylated microtubules in dopaminergic neurons of the midbrain (Feng, 2006).

Like PD and AD, neuronal damage in ALS has also been linked to altered cytoskeletal properties, axon transport, and axonal degeneration (Peters et al., 2015) with altered acetylation of microtubules of particular importance (Gal et al., 2013). Over-expression of mutant SOD1 in animal models has been proposed to alter the acetylation of microtubules by interacting with the HDAC6 enzyme (Gal et al., 2013). One study in three SOD1 transgenic mouse models (A4V, G93A, and G85R) showed that mutant SOD1 and HDAC6 formed a complex, which promoted intraneuronal aggregation of HDAC6, hindering the enzymes’ microtubule deacetylation activity and leading to hyperacetylated microtubules in neurons. The hyperacetylated microtubules were related to increased levels of axonal transport and promoted the spread of pathology in the models (Gal et al., 2013). On the contrary, other studies suggest that decreased microtubule acetylation is implicated in mutant SOD1-associated ALS. For example, an *in vitro* study demonstrated that the microtubule-dependent ER-Golgi transport system was impaired by mutant SOD1 due to decreased microtubule acetylation and reduced microtubule stability in cultured motor neuron cells (Soo et al., 2015). Additionally, acetylated tubulin inclusions were found in mutant SOD1 aggregates (Soo et al., 2015). Other aggregated proteins present in the cytoplasm of induced pluripotent stem cells (iPSCs) derived from ALS patients, including TDP-43 and FUS, have also been linked with altered HDAC6 activity leading to impaired microtubule stability, ER vesicle dynamics, and mitochondria-dependent axonal transport (Fiesel et al., 2010; Guo et al., 2017; Naumann et al., 2018). Fiesel et al. (2010) investigated the effects of altered TDP-43 activity on HDAC6 and microtubule stability. They silenced the TDP-43 gene in both HEK293E and SH-SY5Y cell lines to simulate the disease condition where the nuclear activity of TDP-43 is downregulated and showed that this resulted in reduced HDAC6 mRNA and protein synthesis which was confirmed by the presence of hyperacetylated tubulin (Fiesel et al., 2010). Another study examined iPSC-derived motor neurons from ALS patients with point mutations in the FUS gene, demonstrating impaired axonal transport and lowered ER vesicle transportation which was rescued by HDAC6 inhibitors (Guo et al., 2017).

Altered acetylation of microtubules and resultant alterations in axonal transport is also implicated in HD pathogenesis (Mac Donald et al., 2003; Gauthier et al., 2004; Lee et al., 2004). Trushina et al. (2003) reported increased microtubule deacetylation after binding of mutant huntingtin protein with microtubules in primary neuronal culture models and transgenic mice (Trushina et al., 2003). Altered acetylation of microtubules in HD has been linked with impaired transport of brain-derived neurotrophic factor (BDNF), along with decreased recruitment of motor complexes such as dynein/dynactin and kinesin-1 to microtubules in studied primary cell culture and transgenic HD mouse models (Dompierre et al., 2007). Such deficits could be rescued by pharmacological intervention to increase microtubule acetylation and prevent neuronal damage (Dompierre et al., 2007). Furthermore, *in vitro* studies have also linked hyperacetylated microtubules with reduced vulnerability of striatal cells in HD, by improving autophagic flux and preventing mutant huntingtin diffusing into the neurons (Guedes-Dias et al., 2015).

Altered acetylation in microtubule-associated transport has been linked to a number of other neurodegenerative diseases such as Charcot–Marie–Tooth disease (CMT) and Rett syndrome (RTT) (Xu et al., 2014; Picci et al., 2020). A recent study of an inducible CMT type-2A (CMT2A) mutant MFN2^R94Q^ mouse model with progressive motor and sensory neuronal degeneration demonstrated decreased acetylated α-tubulin in the distal sciatic nerves (Picci et al., 2020). Additionally, CMT type-2D (CMT2D) has been shown to have hypoacetylated α-tubulin-related axonal transport deficits in human stem cell models with the mutant glycyl-tRNA synthetase protein (gene mutation linked to CMT pathophysiology) found to bind to HDAC6 and increase its activity, causing a subsequent reduction in acetylated α-tubulin in peripheral nerves (Smith et al., 2022). The dynamin 2 gene (another gene mutation linked to CMT pathophysiology) is also a microtubule-associated protein, and mutations in the encoded protein have been shown to alter microtubule acetylation in patient-derived cells of CMT. Mutations of dynamin 2 resulted in the altered formation of the Golgi apparatus and microtubule-dependent transport, causing neurodegeneration and neuropathies (Tanabe and Takei, 2009). In RTT, lower levels of acetylated microtubules were present in cultured neuronal cells derived from the *MeCP2* knock-out transgenic RTT mouse model, again altering BDNF vesicle transport, which was linked to altered dendritic growth and synaptic activity (Xu et al., 2014). Lebrun et al. (2021) recently linked loss of motor function and seizures in the *Mecp2*^308/y^ transgenic RTT mouse model, where altered *MeCP2* levels either caused overexpression of HDAC6 or impaired expression of microtubule-associated proteins, which was rescued with HDAC6 inhibitors (Lebrun et al., 2021). Another study used a *MeCP2* deficient cell culture model which also resulted in overexpression of HDAC6, decreasing microtubule acetylation and reducing the structural stability of cilia (Frasca et al., 2020), and impairing the cilium-related Sonic Hedgehog signaling cascade pathway, which is required for forebrain development, The phenotype was improved by using HDAC6 inhibitor, tubacin (Frasca et al., 2020).

### Role of altered acetylation in glial cells in neurodegenerative diseases

While there is a wide base of literature surrounding the role of acetylation in neurons during NDD, altered acetylation has been also studied in glial cells, and has been associated with neurodegeneration. Microglia have been implicated in the progression of several neurodegenerative diseases through inflammatory responses, synapse loss, failure to clear protein aggregates, or by activating neurotoxic astrocytes (Hansen et al., 2018; Madore et al., 2020). Studies have demonstrated the regulatory role of acetylation in the activation of microglia and their inflammatory responses in neurodegenerative diseases. For example, the pan HDAC inhibitor trichostatin A (TSA) potentiated lipopolysaccharide-induced inflammatory responses of microglia in murine N9 and rat primary cultured cells (Suuronen et al., 2003). On the contrary, in a transgenic *Cx3cr1CreERT2 Hdac1^fl/fl^Hdac2^fl/fl^* mouse model, deletion of HDAC1 and HDAC2 from microglial cells enhanced microglial phagocytic activity, which aided in clearing amyloid plaques and improved cognitive function (Datta et al., 2018). Another study demonstrated that acetylated and phosphorylated STAT3 (transcription factor) further activated the studied primary microglial cells (Eufemi et al., 2015).

Additionally, astrocytes have been shown to be regulated through post-translational acetylation processes. In a glial cell culture model, HDAC inhibitors such as TSA upregulated release of neurotrophic factors including GDNF and BDNF from astrocytes followed by protecting dopaminergic neurons (Chen et al., 2006; Wu et al., 2008). On the other hand, SIRT2 inhibitor AGK2 reduced the astrocyte activation level as well as pro-inflammatory factors in a primary cell culture AD model (Scuderi et al., 2014). These data indicate the complex regulatory function of acetylation in glial cells in both healthy and disease states (Neal and Richardson, 2018).

## The future of PTMs as therapeutics for neurodegenerative disease

In this review, we have discussed the vital role of acetylation in the nervous system, and how dysregulation can contribute to the pathogenesis or degeneration seen in neurodegenerative disease. Although we are still in the early stages of research into therapeutics targeting HATs, HDAC inhibitors (HDACi) have been developed in both the preclinical and clinical settings for several years. Treatments targeting HDACs have been widely implicated in cancer therapies, possibly due to an imbalance in acetylation levels closely related to the occurrence of cancers (Mohamed et al., 2007; Chen et al., 2013; Hashimoto et al., 2013; Xu et al., 2019; Wu et al., 2020). Indeed, multiple pan-HDACi have been approved by the FDA for the treatment of cancer, including vorinostat/suberoylanilide hydroxamic acid (SAHA; Marks and Breslow, 2007), belinostat (PXD-101; Foss et al., 2015), and panobinostat (LBH589; Moore, 2016). SAHA was the first FDA-approved HDACi in 2006 and has been successful in treating T-cell lymphomas by reducing the expression of mutant regulatory proteins such as oncogene mutant p53 (Foggetti et al., 2019). Additionally, both pan-HDACi belinostat and panobinostat have been used to treat cutaneous T-cell lymphoma and myeloma (Eckschlager et al., 2017).

Given the success of HDACi in cancer treatment, researchers have sought to repurpose these FDA-approved drugs for neurodegenerative disease, with some FDA-approved HDACi being effective for treating models of HD (Hockly et al., 2003), ALS, PD, and AD (Athira et al., 2021). However, due to the broad range of pan-HDACi targets within cells, more targeted HDACi are being tested pre-clinically such as the HDAC6 specific molecules like ACY-738 or tubastatin A. ACY-1215/Ricolinostat was one of the first HDAC6 specific inhibitors to enter clinical trials for myeloma, lymphoma and metastatic breast cancer (Vogl et al., 2017; Silva et al., 2020; Amengual et al., 2021). The same compound is also in phase II clinical trials for diabetic neuropathy (NCT03176472) and CMT (Benoy et al., 2017). Recently discovered HDACi such as EVP-0334, RDN-929, and CKD-504 are under different phases of clinical trials for the treatment of FTLD, AD, PD, and HD (Rodrigues et al., 2020), and similar compounds such as ACY-738 and Tubastatin A have also shown promising results ameliorating the disease progression in preclinical studies of AD, ALS-FUS, and MS (Multiple Sclerosis) (Kim C. et al., 2012; Guo et al., 2017; LoPresti, 2019). Despite the promise shown by HDACi in treating neurodegenerative diseases, a few caveats need to be considered, including the use of therapies that can cause global hypo or hyper-acetylation in the nervous system.

Although many studies show hypoacetylation to be critical to neurodegeneration processes (Saha and Pahan, 2006), other studies have demonstrated that hyperacetylation is implicated in the pathogenesis of neurodegenerative diseases (Bonet-Ponce et al., 2016; Sohn et al., 2016). In the healthy nervous system, acetylation is highly redundant and exquisitely regulated, however, the use of HAT modifying enzymes or HDACi may lead to off-target effects. The use of HDACi like sodium butyrate has led to hyperacetylation of histone H4 which subsequently facilitated the expression of an oxidative stress-sensitive protein known as PKCδ and caused neurotoxicity in human dopaminergic neuronal cells (Jin et al., 2014). Additionally, oxidative stress molecules that are produced during the progression of these neurodegenerative diseases have been shown to hyperacetylate microtubules and disrupt autophagic trafficking in studied ARPE-19 cells which were exposed to Rotenone drug to induce oxidative stress. This stress-induced microtubule hyperacetylation was successfully reduced by using free radical scavenger drugs like N-acetylcysteine (Bonet-Ponce et al., 2016). Further studies are required to elucidate whether hypoacetylation or hyperacetylation has therapeutic benefits for axons in neurodegenerative diseases.

There are several hurdles to overcome for the use of HDACi in treating neurodegenerative diseases, that focus on two core themes: target preference (developing HDACi for selective isoforms or families), and selective delivery (the ability to target specific tissue or cell type for therapy). Small molecule inhibitors, such as ACY molecules, ACY-738, and ACY-1215 are prime examples of target preference and specificity, as both compounds have been reported to be HDAC6-specific inhibitors (Santo et al., 2012). However, their specificity depends greatly on the tissue uptake and overall dose. For example, high levels of ACY-738 have been reported to alter H3 acetylation levels in mesangial cell lines and the spinal cord of WT mice (Regna et al., 2015; Rossaert et al., 2019). Target specificity also remains a caveat in the use of PTM modifying drugs in the clinic for neurodegenerative diseases. For instance, reports have suggested that HDAC6i ACY-738 increased the life span by 41 days in transgenic *FUS*+/+ HDAC6 knock-out mice, suggesting potential off-target activity of ACY-738 drug (Rossaert et al., 2019). Additionally, some other off-target effects of these inhibitors can be cellular apoptosis or T-cell-mediated immune responses (Majid et al., 2015).

Further difficulty for potential drug candidates of NDD is the ability for novel small molecule inhibitors to cross the blood brain barrier (BBB). HDACi such as MS-275, SAHA, valproic acid and Tubastatin A showed low BBB permeability in studies (Choi et al., 2019). As a result, achieving the required therapeutic efficacy requires higher dosing, which can lead to potential off-target effects (Choi et al., 2019). Literature suggests that benzylic amide derivatives showed higher BBB permeability and inhibitory activity in the baboon model against HDAC1 and 2. The BBB permeability of these drug derivatives were optimized through image guided synthetic process (Seo et al., 2014). Another recent study designed benzoheterocycle derivatives that were structurally different from the available HDACi and identified benzothiazole derivative 9b which showed higher BBB permeability than SAHA. These approaches for developing HDACi should be further studied to achieve the desired therapeutic treatment for neurodegenerative diseases (Choi et al., 2019). Further refinement of selectivity and delivery methods remain to be developed. To overcome the limitations of tissue specificity and off-target effects of HDACi, advanced genomic targeting methodologies such as CRISPR-Cas has been used to control targeted HAT/HDAC activity. Studies have successfully fused dCas9 to the p300 acetyltransferase to catalyze acetylation of H3 followed by transcriptional activation of the targeted genes (Hilton et al., 2015; Shrimp et al., 2018), however, methods for cell-type specific treatment remain to be developed.

## Conclusion

In conclusion, acetylation plays a critical role in maintaining the homeostasis of cellular proteins and cytoskeleton. This tightly regulated process has been shown to be dysregulated in neurodegenerative disease; however, the underlying pathophysiological mechanisms are yet to be fully understood. Nevertheless, through emerging therapeutics, altered acetylation can be a promising target to limit or prevent the pathological processes that lead to protein aggregation or defects of axonal transport in neurodegenerative diseases.

## Author contributions

FK, RA, AP, and AK: conceptualization. FK: writing - original draft. FK, RA, AP, AC, and AK: writing - reviewing and editing. All authors contributed to the article and approved the submitted version.

## Funding

This work was supported by FightMND, MND Research Australia, the National Health and Medical Research Council of Australia, and the JO and JR Wicking Trust (equity trustees). AK NHMRC fellowship number is APP1136913.

## Conflict of interest

The authors declare that the research was conducted in the absence of any commercial or financial relationships that could be construed as a potential conflict of interest.

## Publisher’s note

All claims expressed in this article are solely those of the authors and do not necessarily represent those of their affiliated organizations, or those of the publisher, the editors and the reviewers. Any product that may be evaluated in this article, or claim that may be made by its manufacturer, is not guaranteed or endorsed by the publisher.
